# Electrodeposition of Silver Nanoparticles on Indium-Doped Tin Oxide Using Hydrogel Electrolyte for Hydrogen Peroxide Sensing

**DOI:** 10.3390/nano13010048

**Published:** 2022-12-22

**Authors:** Jihyeon Kim, Byung-Kwon Kim, Kyungsoon Park

**Affiliations:** 1 Department of Chemistry and Cosmetics, Jeju National University, Jeju 690-756, Republic of Korea; 2 Department of Chemistry and Nanoscience, Ewha Womans University, Seoul 03760, Republic of Korea

**Keywords:** silver nanoparticle, electrodeposition, hydrogel, electrochemical H_2_O_2_ sensing

## Abstract

Nanoparticles are used in various fields, including fuel cells, energy conversion devices, and sensors, because of their large surface area and excellent catalytic properties. Although various methods of synthesizing nanoparticles are available, the most popular is the solution-phase reduction of metal ions. Electrodeposition is a method of reducing metal ions in solution and is widely used because of its various advantages. In this study, Ag nanoparticles with a narrow size distribution were evenly dispersed on the surface of an electrode by applying electrodeposition in an agarose hydrogel medium instead of in solution, confirming the feasibility of Ag deposition in agarose hydrogel, even at a lower reduction potential than that in solution. These results are attributed to the electrolyte effect owing to the hydrophilic backbone of the agarose hydrogel and the gel effect, which reduces unexpected convection. H_2_O_2_ was detected by using the Ag nanoparticles synthesized in agarose hydrogel, and the limit of detection for H_2_O_2_ was found to be 4.82 µM, with a dynamic range of 1–500 µM. The nanoparticle synthesis platform proposed in this study is expected to be actively used for the synthesis of other metal/nonmetal nanoparticles.

## 1. Introduction

Nanostructured metals deposited on diverse substrates have played a pivotal role in the development of technological applications, including microelectronics [1,2], sensing [3,4], electroplating [5], energy storage [6,7], and the enhancement of Raman scattering [8] and electrocatalysis [9,10]. Metal nanoparticles have a high surface-to-volume ratio, with the capability to interact with target molecules, good electrical and catalytic properties, and a large number of active sites [11]. The properties of metal nanoparticles differ from those of the corresponding bulk materials. Among the various strategies for fabricating nanocomposites, electrochemical deposition is a practical alternative to gaseous phase deposition under vacuum, such as vapor deposition, sputtering, chemical vapor deposition [12,13], and in the solution phase, such as printing and dip-coating [14,15]. Electrochemical techniques can be used as not only facile methods for fabricating metal nanoparticles, but also versatile analytical tools for manipulating the electrochemical behavior. The potential applied to the working electrode and the duration can affect important properties of nanoparticles, such as the size, density, morphology, and electrocatalytic effects [16].

Aqueous electrolytes are typically used in conventional electrodeposition processes; however, they have some limitations, including gas evolution, the need for complexing agents, water pollution in industrial use, and irregular deposition caused by natural convection for long-term electrolysis [17,18]. To overcome the limitations of aqueous solvents, several alternative media, such as organic solvents or ionic liquids, have been used, but the toxicity, volatility, and poor solubility of simple metal salts are the major barriers to their application [19,20,21,22].

A hydrogel is a three-dimensional (3D) network comprising a hydrophilic functional group of polymer chains, which can imbibe a large amount of water without dissolving [23,24]. The unique structures and properties of hydrogels provide useful applications in many research areas, including tissue engineering [25], drug delivery [26,27], biosensors [28], and energy storage and conversion fields [29]. Agarose is a widely used material among various types of hydrogels because it is inexpensive, non-toxic, and easy to synthesize and has high structural flexibility. Agarose is a linear polysaccharide consisting of 1,3-linked β-D-galactopyranose and 1,4-linked 3,4-anhydro-α-L-galactopyranose repeating units [30]. Recently, Hwang et al. used a hydrogel pen (HYPER) for the electrodeposition of platinum between pyramid-shaped agarose and a gold substrate by controlling the contact area [31]. We also reported the mass transport properties of solutes on the agarose interface, which precluded the effect of natural convection on the long-term electrochemistry [32].

The selective, rapid, and quantitative determination of hydrogen peroxide (H_2_O_2_) has attracted considerable attention because of the essential role of this compound in the food [33], pharmaceutical [34], clinical [35], cosmetic [36], and biochemical industries [37]. Various analytical methods have been employed to trace H_2_O_2_, including chromatography [38], fluorescence [39], chemiluminescence [40], and electrochemical methods [41,42]. Among these techniques, electrochemical analysis is the most suitable owing to its speed, low detection limit, simple instrumentation, and real-time measurement capabilities. Electrochemical detection of H_2_O_2_ is commonly based on the use of enzymes as signal transducers [43]. Although enzyme-based sensors have an ultra-low detection limit for analytes, their application to H_2_O_2_ is limited because of their inherent poor stability, which causes denaturation. Moreover, the catalytic activity and selectivity for H_2_O_2_ are significantly reduced by other interfering substances with similar detection potentials, such as dopamine, uric acid, catecholamine, and ascorbic acid. Because of these disadvantages of enzyme-based electrochemical sensing for H_2_O_2_, metal nanoparticles are prospectively good alternatives for replacing enzyme transducers owing to their outstanding conductivity, long-term stability, and excellent electrocatalytic activity [41,44].

**Scheme 1:**
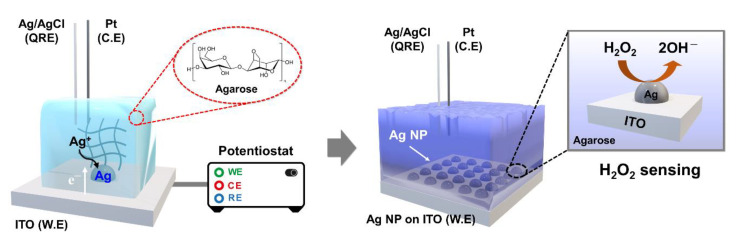
Schematic of cell configuration for the electrodeposition of Ag nanoparticles on ITO and H_2_O_2_ sensing.

Herein, we report a simple method for the electrodeposition of Ag nanoparticles on indium tin oxide (ITO) by using agarose hydrogel media. Agarose-based Ag deposition exhibits numerous desirable characteristics, such as well-dispersed and uniform particles and adjustable properties, compared with the conventional solution-phase process. Electrodeposition is a versatile method of controlling the shape and size of metal nanoparticles on a conducting surface by adjusting the preparation conditions [45,46]. Therefore, the size of the Ag nanoparticles can be controlled by adjusting the deposition time without any interference (i.e., natural convection), as confirmed by performing field-emission scanning electron microscopy (FE-SEM) analysis of the particle morphology. Metal nanoparticles are considered the most promising alternatives to enzymes for electrochemical detection [47]. In this study, the electrocatalytic activity of Ag nanoparticles for H_2_O_2_ sensing is evaluated using an electrochemical method. Many methods of fabricating metal nanoparticles in bulk solutions are known and widely used; however, hydrogel-based methods of synthesizing nanocomposites have rarely been studied. Therefore, our approach is expected to be a good candidate for the synthesis of various types of metal nanoparticles. The electrochemical setup for Ag deposition and H_2_O_2_ sensing using agarose hydrogel are depicted in Scheme 1.

## 2. Experimental

### 2.1. Materials

Agarose (Low EEO), silver nitrate (AgNO_3_, 97%), potassium perchlorate (KClO_4,_ 99.9%), and nitric acid (HNO_3_, ~70%) were obtained from Sigma-Aldrich (St. Louis, MO, USA). Perchloric acid (HClO_4_, 70%) and hydrogen peroxide (H_2_O_2_, 30%) were purchased from Acros Organic and Fisher Chemical, respectively. Silver was electrodeposited on indium tin oxide (ITO)-coated glass (Omniscience, Yongin-si, Republic of Korea, 10 Ω cm^−2^ sheet resistance). The ITO was cleaned with acetone and isopropanol in an ultrasonic bath for 5 min and dried under a N_2_ stream. All chemicals and reagents used in this work were of reagent grade. Water (>18 MΩ·cm) was obtained from a Millipore Milli-Q purification system.

### 2.2. Preparation of Agarose Hydrogel for Electrochemical Measurement

The agarose solutions (3.2 wt%) were prepared in a microwavable cylindrical container equipped with a sealing cap. The solution in the container was heated under microwave (700 W power) until the agarose was completely dissolved. The dissolved solution was placed in a vacuum desiccator to remove air bubbles that disrupted the gel. The prepared agarose solution (viscous and transparent) was poured into a glass mold and cooled slowly in a humidity-controlled chamber. The solidified agarose was cut into the desired sizes, carefully separated from the glass mold, and stored in distilled water. Before the electrochemical measurements, the agarose gels were immersed in an aqueous solution of redox molecules and a supporting electrolyte for 12 h to achieve complete equilibrium. For the electrochemical measurements, a redox material containing agarose gel was installed in a homemade cell.

### 2.3. Electrochemical and FE-SEM Measurement

Electrochemical measurements were performed in a conventional three-electrode cell using a CHI 601e potentiostat (CH Instruments, Austin, TX, USA). Indium tin oxide (ITO; 2.5 × 2.5 cm) was used as the working electrode, and a Pt wire and Ag/AgCl wire were used as the counter electrode and quasi-reference electrode, respectively, and were positioned inside the agarose gel. A homemade cell equipped with a vertical press was used to ensure conformal contact between the agarose hydrogel and ITO electrode. The surface morphology of the electrodeposited Ag on ITO was characterized by using FE-SEM (TESCAN, MIRA III, 15 kV, WD = 9.9~10.3 mm) which was equipped with detector for imaging of secondary electron. Additionally, the chemical composition were analyzed by the FE-SEM equipped with energy dispersive spectrometry (EDS, Oxford Instruments, Oxford, UK).

### 2.4. Electrodeposition of Ag on ITO and Electrochemical H_2_O_2_ Reduction

The electrochemical deposition of Ag on ITO is potentiostatically similar to that in the aforementioned cell configuration. Agarose gel was immersed in a mixture of 1 mM AgNO_3_, 5 mM KClO_4_, and 1 mM HClO_4_ as a soaking solution which was adjusted to pH 4 (see Figure S1 in the Supplementary Materials). Cyclic voltammetry (CV) scans were recorded in the range of 0.4 to −0.5 V at a scan rate of 50 mV/s. Chronoamperometry (CA) for Ag deposition was performed by applying −0.35 V (vs. Ag/AgCl quasi-reference electrode (QRE)) to the ITO working electrode in contact with the agarose hydrogel. To ensure a conformal contact, constant pressure was applied between agarose hydrogel and ITO by home-made press machine. The electrocatalytic effect of Ag on ITO (Ag deposition time: 10 s, Figure S2) for H_2_O_2_ detection was determined by performing CV and chronocoulometry (CC) in an electrochemical buffer (EB; 0.1 M phosphate buffer and 700 mM NaCl; pH 7.4) with different concentrations of H_2_O_2_.

## 3. Results and Discussion

**Figure 1:**
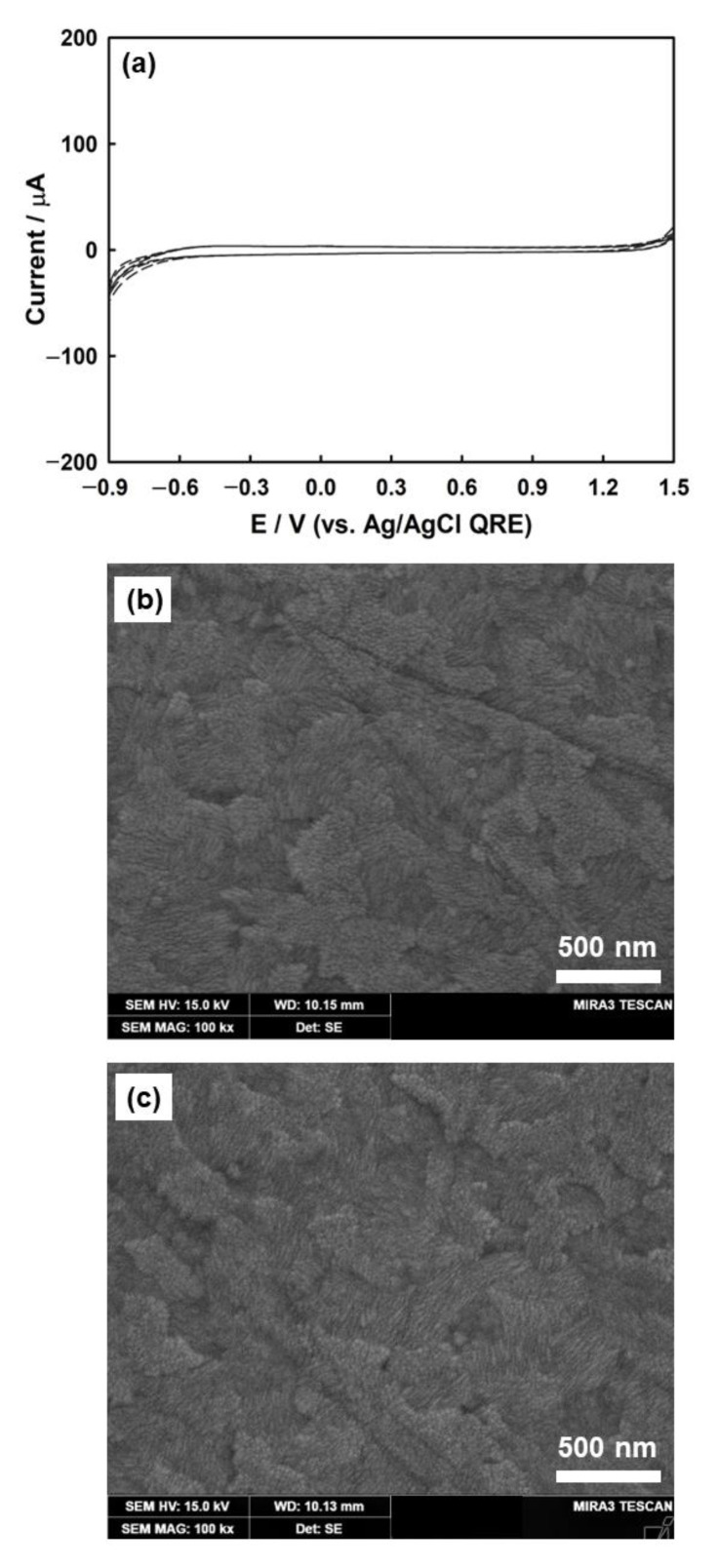
Three cycles of CVs for bare ITO electrode (**a**) in a solution of 1 mM HClO_4_ and 5 mM KClO_4_ (scan rate, *v* = 100 mV/s). SEM image of bare ITO (**b**) before and (**c**) after three cycles of CV.

The scanning electron microscopy (SEM) images show the morphology of the bare ITO electrode before and after three CV cycles. The bare ITO electrode showed densely attached grains with crystalline particles of approximately 30 nm in diameter (Figure 1b). This structure imparted highly conductive properties to the ITO surface. Figure 1c shows the SEM micrograph after three CV cycles, in which the ITO surface remained stable without any damage caused by ITO reduction or oxygen evolution.

ITO was used as the working electrode for the electrodeposition of Ag nanoparticles. Although ITO has low electrical resistivity (~10^−4^ Ω·cm) with high transparency to UV-vis light (>90% transmittance), it is known that electrochemically reduced ITO electrodes turn dark brown in color and are less conductive [48,49]. To prevent this reduction, the ITO electrode was evaluated by employing CV to check its stability within the potential windows for the electrodeposition of silver. Figure 1a shows that ITO was stable in the supporting electrolyte over the potential range of +1.3 V to −0.7 V. ITO was reduced at a potential more negative than −0.7 V, and oxygen evolution was initiated at a potential more positive than +1.3 V.

**Figure 2:**
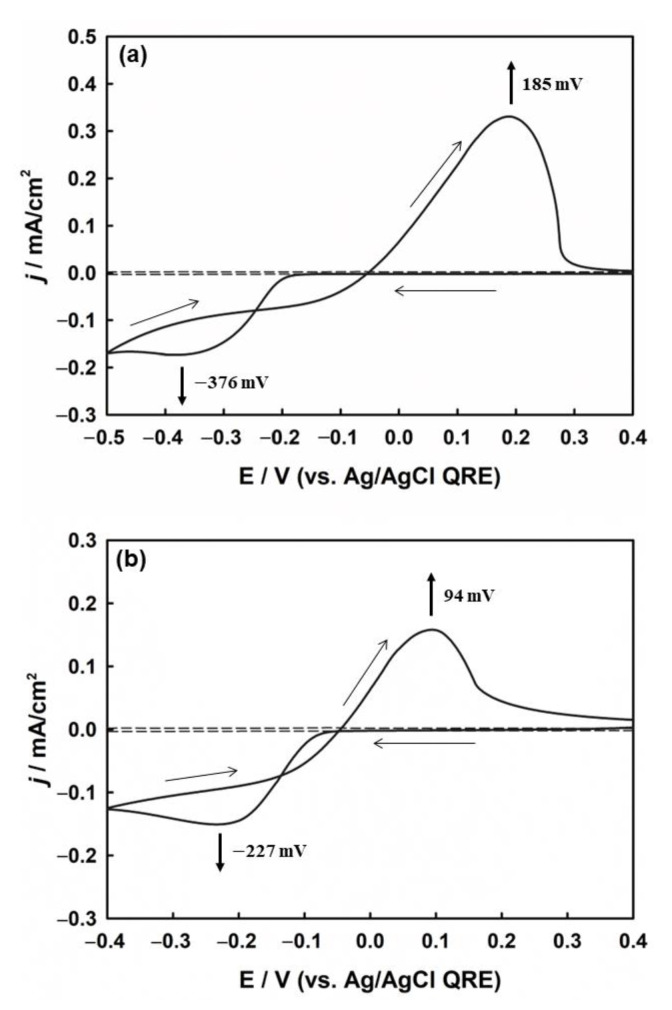
CVs of silver redox process on ITO electrode (**a**) in solution and (**b**) in agarose hydrogel, both containing 2 mM AgNO_3_, 1 mM HClO_4_, and 5 mM KClO_4_. The dotted line represents the background signal without AgNO_3_. Scan rate: 50 mV/s.

The electrochemical process of silver deposition on the ITO substrate was studied using CV in solution and agarose hydrogel (Figure 2). In the solution (Figure 2a), two distinct peaks related to the reduction of silver ions and the oxidation of silver previously deposited on the ITO surface are observed. The sharp increase in the cathodic current at −0.2 V is an indication of an increase in silver nucleation and particle growth, reaching the cathodic peak current (*I_pc_*) of −176 mA/cm^2^ at −0.376 V. The reaction related to the peak current (*I_pc_*) is indicated by Equation (1):

(1) $${\mathrm{Ag}}^{+}+e^{-}\;\to \;\mathrm{Ag}$$

The deposition potential between metal ions and the foreign substrate is generally higher than that between the same metal particles due to crystallographic substrate-metal misfit, except in the case of underpotential deposition [17]. Therefore, the deposition of Ag^+^ on the ITO electrode was more negative than the formal potential of Ag^+^/Ag. However, during the positive scan, the oxidation of silver began from the same silver surface that had already been deposited onto the ITO surface. Owing to the difference in the deposition and stripping potentials, a crossover between the cathodic and anodic current traces occurred. Therefore, crossover is another method of forming metal nuclei on the electrode [50].

Figure 2b shows the cyclic voltammogram for the electrochemical deposition of Ag on the ITO substrate in the agarose hydrogel. From the electrochemical analysis, it was confirmed that the agarose hydrogel was not adsorbed on the surface of the synthesized Ag nanoparticles. (see Figure S3 in the Supplementary Materials). The CV profile was similar to that in solution, but presented different onset potentials for silver deposition. The decrease in both the cathodic and anodic overpotentials compared to those in solution suggests that silver deposition in agarose is more favorable than in solution, which is assumed to be due to the higher interaction and stabilization between charged silver ions and the hydrophilic moieties of the agarose polymer backbone.

**Figure 3:**
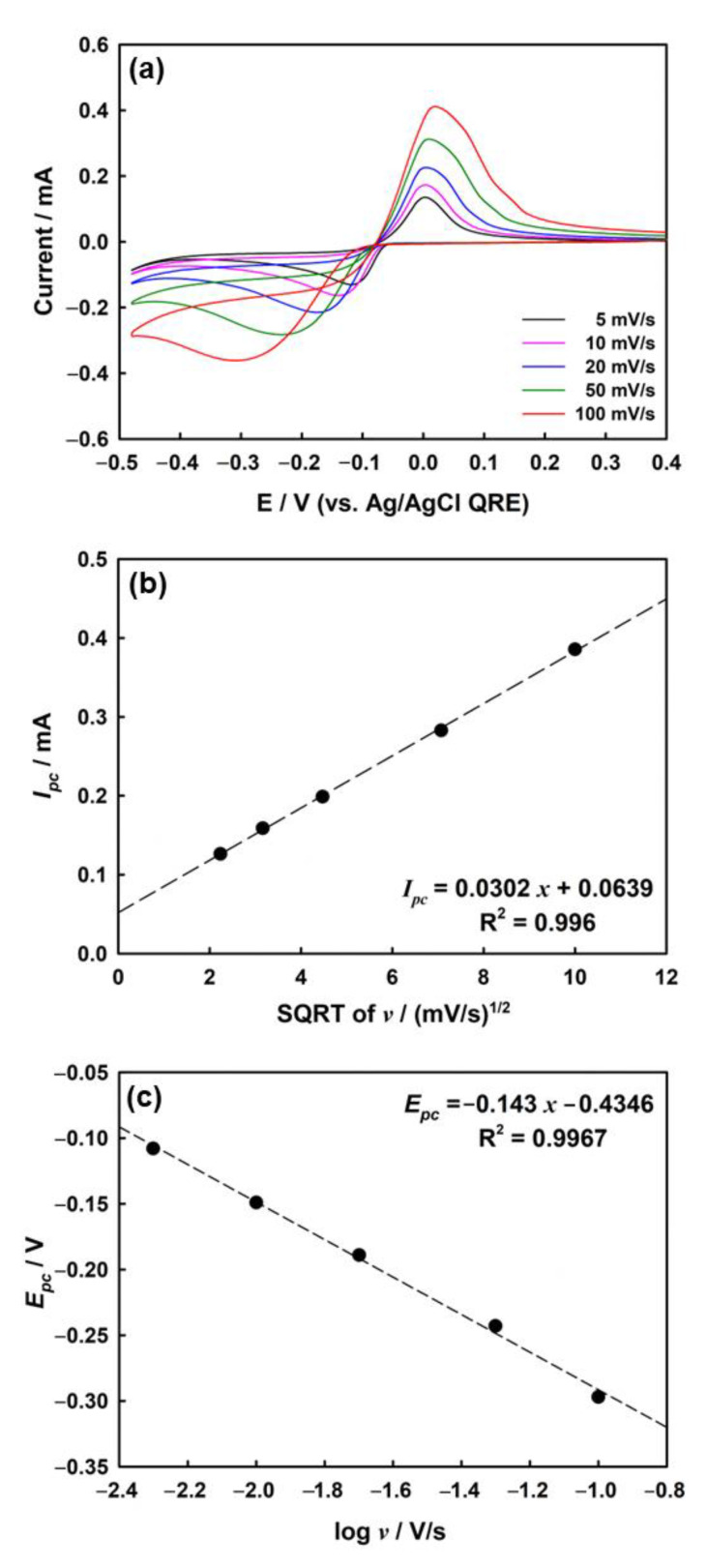
(**a**) CVs of ITO electrode in agarose hydrogel containing 2 mM AgNO_3_, 1 mM HClO_4_, and 5 mM KClO_4_ depending on scan rate. (**b**) Plots of cathodic peak current (*I_pc_*) vs. square root of the scan rate (*v*) and (**c**) dependence of peak potential, *E_pc_*, vs. log *v* in 4.8 wt% of agarose hydrogel.

To confirm the mass transport properties of silver ions in the agarose media, the CV of the silver deposition process was measured by varying the scan rate in the agarose hydrogel. As shown in Figure 3a, the cathodic and anodic currents both increased as the scan rate increased from 5 to 100 mV/s.

A linear relationship between the cathodic peak current (*I_pc_*) and square root of the scan rate (*v*) was observed for the silver deposition process (Figure 3b). Linearity is generally observed in electrochemical reactions in which the mass transport of redox molecules is mostly governed by the diffusion process [51]. Therefore, the main factor affecting mass transfer in agarose hydrogels is diffusion, whereas other factors (i.e., convection and migration) are not worth considering at normal scan rates [32].

Figure 3c shows the change in the cathodic peak potential (*E_pc_*) according to the log (scan rate, *v*) for the electrodeposition of silver on ITO in agarose hydrogel. The coefficient of charge transfer (*α*) is calculated to be 0.38 at room temperature (298 K) by using Equation (2) [52,53]:

│*E_p_ – E_p/_*_2_│ = 1.857*RT*/*αnF* (2)

where *R* is the gas constant, *T* is the absolute temperature, *α* is the charge-transfer coefficient, *n* is the number of electrons in the rate-determining step, and *F* is the Faraday constant.

**Figure 4:**
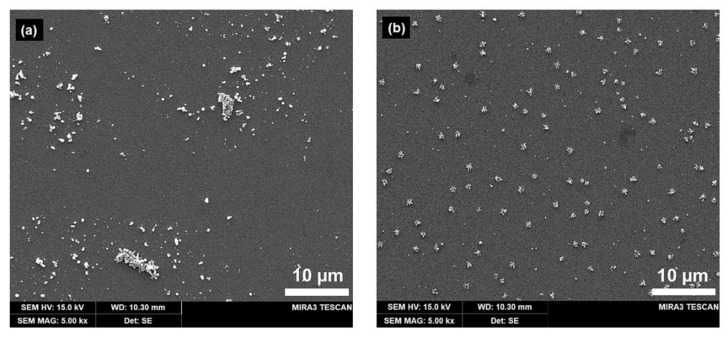
SEM images of silver clusters electrodeposited on ITO electrode (**a**) in solution, (**b**) in agarose hydrogel (3.2 wt%) from a solution containing 1 mM AgNO_3_, 1 mM HClO_4_, and 5 mM KClO_4_. Electrodeposition was performed at −0.35 V (vs. Ag/AgCl quasi-reference electrode (QRE)) for 10 s.

Silver was electrodeposited on the ITO electrode under two conditions: in solution and agarose hydrogel. Figure 4a show the SEM images of the Ag deposits formed at −0.35 V (vs. Ag/AgCl QRE) over 10 s in solution, demonstrating a broad range of sizes (refer Figure S4a and Figure S5a in the Supplementary Materials) and irregular shapes. This suggests that controlling the morphology or distribution of the deposited nanoparticles is difficult in the solution phase, which is assumed to be caused by uncontrollable mass transport properties (i.e., natural convection). Ag deposition in the agarose hydrogel (refer Figure S4b and Figure S5b in the Supplementary Materials) afforded nanoparticles with a regular shape, which were well-distributed laterally compared to those obtained via solution-phase electrodeposition (Figure 4b). Additionally, the total amount of Ag nanoparticles on ITO was approximately 7.265 × 10^−12^ mol/cm^2^ using anodic stripping voltammetry (see Figure S6 in the Supplementary Materials). We assumed that the interaction between the Ag^+^ ions and the hydrophilic polymer network of the hydrogel can reduce natural convection [32], which causes irregular transport of the solute to the electrode surface. The transport of metal ions to the electrode surface is crucial for the electrodeposition of metal nanoparticles. The pore structure of the agarose hydrogel could be controlled by varying its concentration. From these results, the agarose hydrogel appeared not only to be a good template, but also a well-controllable reaction medium for the electrochemical deposition of metal nanoparticles.

The influence of the duration of application of the potential (hereinafter, potential duration) on silver deposition in the agarose hydrogel was determined by varying the deposition time using a ITO working electrode. Small metal particles have adjustable properties (e.g., magnetic, optical, and electronic) that can be tuned based on the structure, interparticle density, and particle size. In a typical electrodeposition process, the potential duration applied to the electrodes influences the nucleation and crystal growth of the metal nanoparticles [17,46].

**Figure 5:**
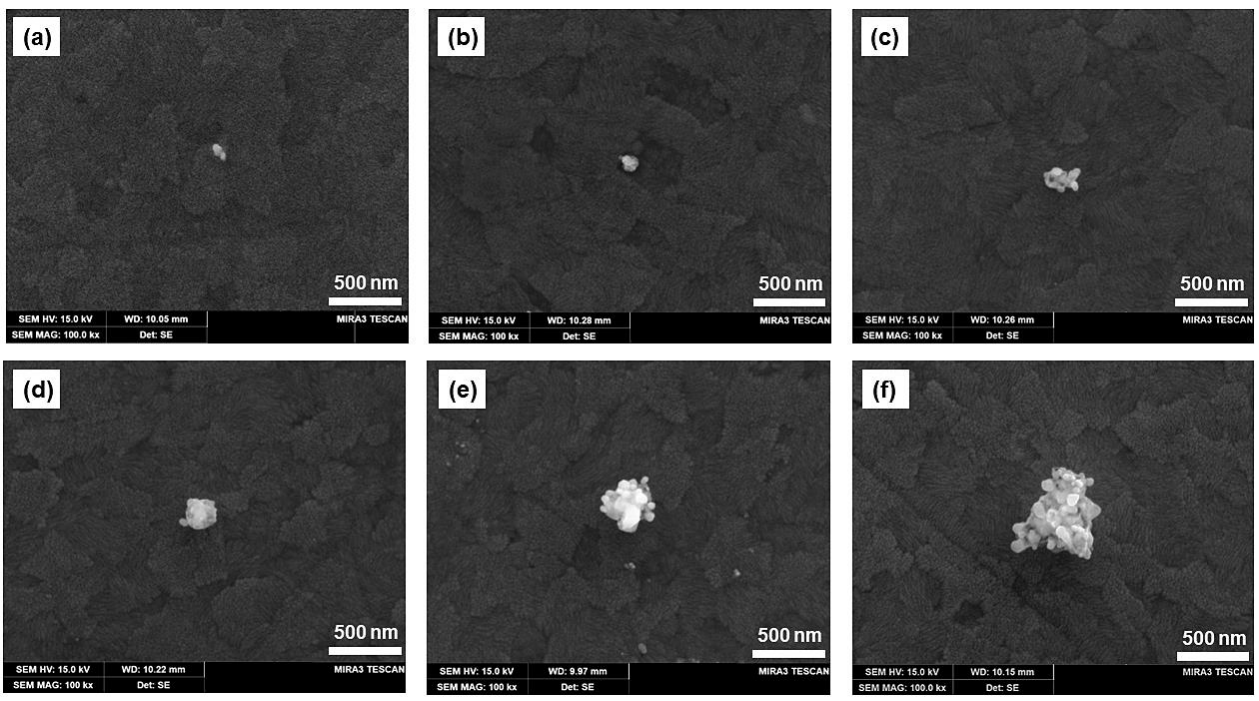
SEM micrographs of silver nanoparticles deposited on ITO electrode in agarose hydrogel containing 1 mM AgNO_3_ at a potential of −0.35 V at deposition times of (**a**) 0.5 s, (**b**) 1.0 s, (**c**) 1.5 s, (**d**) 2.0 s, (**e**) 5.0 s, and (**f**) 10.0 s.

Figure 5 shows the SEM micrograph of the Ag-deposited ITO electrode surface when a potential of −0.35 V against the Ag/AgCl QRE is applied for durations of 0.5 to 10 s in the silver-containing agarose hydrogel. The SEM images show marked differences in the size and morphology of the particles with an increasing deposition time. As shown in Figure 5a, after electrodeposition of 1.0 mM AgNO_3_ for 0.5 s, silver deposits with a diameter less than 100 nm are obtained. This indicates that the onset of silver nucleation occurs earlier than 0.5 s. Although uneven, at longer deposition times, the size of the silver deposits increases owing to the aggregation of small clusters (Figure 5a–f). Precise control of the size and morphology is beyond the scope of this work, and the well-controlled fabrication of electrodeposited nanoparticles is still a challenging technique that depends on various parameters. Nonetheless, Ag nanomaterials of various sizes were successfully fabricated on the ITO electrode surface in agarose hydrogel by controlling the deposition time.

Silver particles are known to act as electrocatalysts for H_2_O_2_ reduction [52]. H_2_O_2_ is reduced on the electrode via the following mechanism [54]:

(3) $$H_{2}O_{2}\;{}_{\left(\mathrm{aq}\right)}\;+\;e^{-}\;\to \;{\mathrm{OH}}_{\mathrm{ads}}\;+\;{\mathrm{OH}}^{-}{}_{\left(\mathrm{aq}\right)}$$

(4) $${\mathrm{OH}}_{\mathrm{ads}}\;+\;e^{-}\;\to \;{\mathrm{OH}}^{-}{}_{\left(\mathrm{aq}\right)}$$

The net reduction of H_2_O_2_ in solution is given as

(5) $$H_{2}O_{2}\;{}_{\left(\mathrm{aq}\right)}\;+\;2e^{-}\;\to \;2{\mathrm{OH}}^{-}{}_{\left(\mathrm{aq}\right)}$$

**Figure 6:**
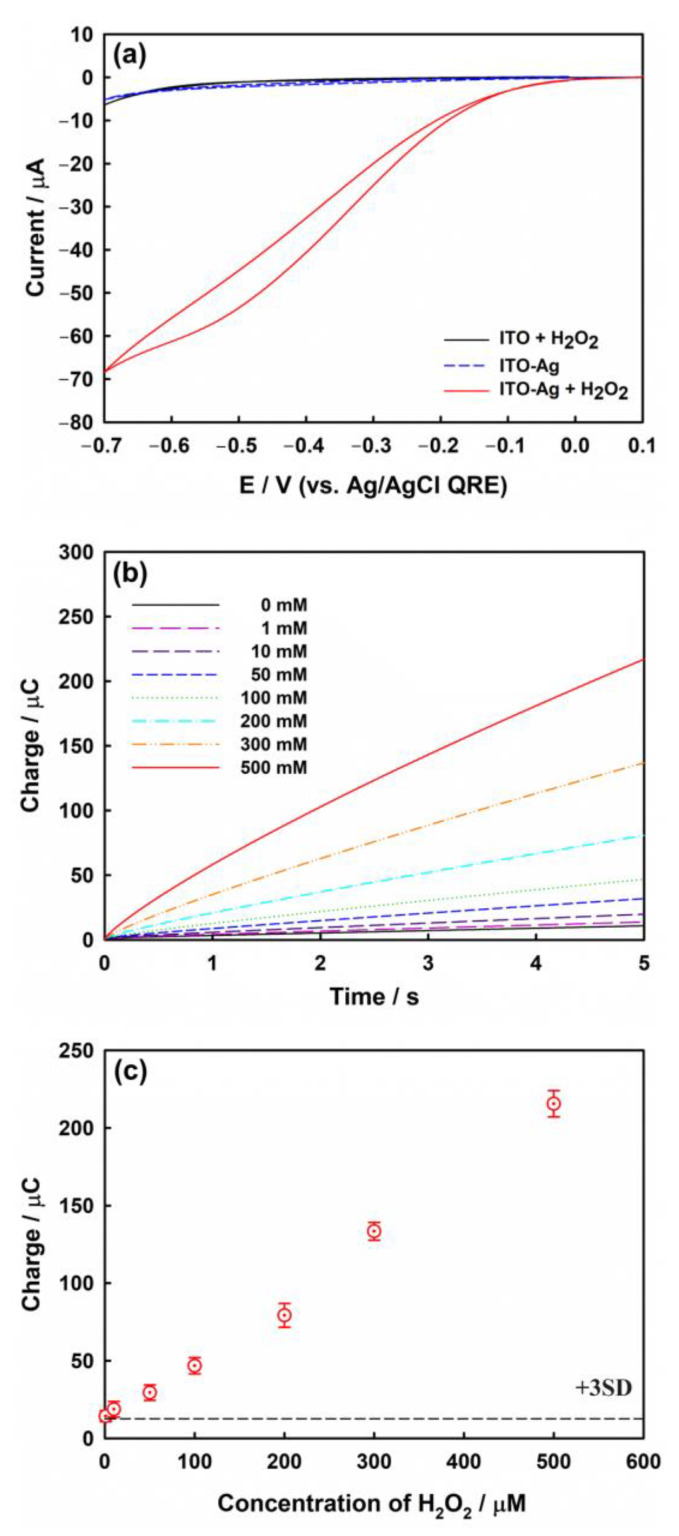
(**a**) CVs obtained with bare ITO (black line) and ITO electrode modified with Ag nanoparticles in the presence (red line) or absence of 1.0 mM H_2_O_2_ (blue dotted line) in phosphate buffer (pH 7.4). Scan rate: 100 mV/s. (**b**) CCs obtained at −0.6 V in phosphate buffer (pH 7.4) containing various concentrations (1, 10, 50, 100, 200, 300, and 500 μM) of H_2_O_2_. (**c**) Calibration plot for the charge values at 5 s in panel (**b**). The error bars represent the standard deviation (SD) of three independent measurements. All data were corrected by the mean value of the double layer charging current determined from five measurements at zero concentration of H_2_O_2_. The dotted line shows three times the SD of the charging current obtained at zero concentration of H_2_O_2_. Ag deposition condition: potential of −0.35 V (vs. Ag/AgCl QRE) for 10.0 s.

To confirm the electrocatalytic properties of the prepared Ag nanostructures, the electroreduction of H_2_O_2_ was characterized by employing CV within the stable potential range of the ITO electrode in a deaerated 0.1 M phosphate-buffered solution (pH 7.4) containing 1.0 mM H_2_O_2_, at a scan rate of 100 mV/s (Figure 6a). The current response of the bare ITO electrode (black line) for H_2_O_2_ is small, indicating negligible electrocatalytic activity. In the absence of H_2_O_2_ (blue dotted line), even the ITO electrode modified with Ag nanoparticles exhibits a weak current, similar to that of bare ITO without H_2_O_2_. In both cases, the H_2_O_2_ reduction signals are negligible. However, the strongest catalytic reduction current is observed for the Ag-modified ITO electrode in the presence of H_2_O_2_ at −0.6 V vs. Ag/AgCl QRE. This suggests that H_2_O_2_ can be reduced only on the Ag surface of the ITO electrode, and the Ag-ITO electrode possesses excellent electrocatalytic capability for H_2_O_2_ reduction.

To investigate the analytical application of the modified electrode for H_2_O_2_ sensing, chronocoulograms were acquired for the Ag-modified ITO electrodes using different concentrations of H_2_O_2_ (Figure 6b). In chronocoulometry (CC) [55], the integrated current at a given period of time is used as the sensing signal. These signals show superior reproducibility over those obtained by using potential sweep techniques such as CV. The capacitive charging current, as the background level in CV, can be a major obstacle in electrochemical sensing, whereas the effect of the capacitive charging current in CC can be sufficiently reduced by the integration process under sufficiently long-term measurements. The typical charge-time response is proportional to the H_2_O_2_ concentration in the range of 1.0–500 μM. The mean charge value at 5.0 s in the absence of H_2_O_2_ is 10.7 μC, and the standard deviation (SD) is 0.65 μC. Figure 6c shows the calibration plot for the charge data obtained at 5.0 s presented in Figure 6b. The estimated limit of detection (LOD) using Equation (6) for this sensing platform is ca. 4.83 μM.

LOD = 3*S_b_*/*m* (6)

where *S_b_* is the SD of the blank and *m* is the slope of the corresponding calibration curve (refer Figure S7 in the Supplementary Materials) [56]. To confirm the selectivity of our sensor system, amperometric *i-t* response was obtained in the presence of interference molecule such as nitrate, glucose, and ascorbic acid (refer Figure S8 in the Supplementary Materials). These results reveal that the developed hydrogel-based Ag-ITO sensor exhibits excellent performance in terms of high sensitivity and selectivity as well as low detection limit, comparable to those of other non-enzymatic H_2_O_2_ sensors (see Table 1).

## 4. Conclusions

In this study, we successfully deposited Ag nanoparticles on an ITO electrode in agarose hydrogel. Electrodeposition of Ag nanoparticles in agarose hydrogels has the following advantages over deposition in solution: (1) nanoparticles can be deposited with relatively mild reduction potentials; (2) nanoparticles are evenly dispersed and deposited on the electrode surface; (3) the sizes of the synthesized nanoparticles are relatively homogeneous. These advantages may be attributed to the hydrophilic backbone of the agarose hydrogel and the minimization of unexpected convection. Ag nanoparticles synthesized in agarose hydrogel, as a solid electrolyte, showed good sensing ability for H_2_O_2_. The LOD of our sensing platform for H_2_O_2_ detection was 4.83 µM, with a dynamic range of 1–500 µM. The LOD and dynamic range were extremely low compared with those observed in several recent studies. The proposed method for nanoparticle electrosynthesis in agarose hydrogel is not limited to Ag. Agarose hydrogels can be actively used for the synthesis of various metal and non-metal nanoparticles and are expected to be used as important nanoparticles or nanocatalyst synthetic media.

## Figures and Tables

**Table 1:** Comparison of various non-enzymatic H_2_O_2_ sensors based on nanomaterials.

Sensor Type	LOD (μM)	Dynamic Range (mM)	Ref.
Cu NPs@GCE	3.4	8–70	[57]
Co_3_O_4_ NWs@rGO	2.4	0.015–0.675	[58]
Ag-mSiO_2_ NPs@GCE	3.00	0.004–10	[44]
C_rod_@Ag-Ps	67	0.5–5.0	[59]
AuNPs-NH_2_/Cu-MOF/GCE	1.2	0.005–0.85	[60]
Ni/Ag@C	10	0.03–17	[61]
AgNPs@rGO@PANI	0.05	0.00001–1	[62]
AuCu alloy NPs Ag NPs@ITO	10.93 4.83	0.05–10 0.001–0.5	[63] This work

NP—nanoparticle, GCE—glassy carbon electrode, NW—nanowire, rGO—reduced graphene oxide, MOF—metal-organic framework, PANI—polyanlinie, MWCNT—multi-walled carbon nanotube.

## Data Availability

Not applicable.

## Supplementary Materials

The following supporting information can be downloaded at: https://www.mdpi.com/article/10.3390/nano13010048/s1, Figure S1: Cyclic voltammetry of silver deposition on ITO using agarose hydrogel (3.2 wt%) as function of pH in 2 mM AgNO_3_, 1 mM HClO_4_, and 5 mM KClO_4_. pH was adjusted by HNO_3_. Scan rate: 10 mV/s; Figure S2: The calibration plot for the H_2_O_2_ oxidation charge values of chronocoulograms obtained at −0.6 V according to the Ag deposition time. The H_2_O_2_ sensing experiment conducted in 0.1 M phosphate buffer (pH 7.4) and 700 mM NaCl with 300 μM H_2_O_2_. The error bars represent the three independent measurements; Linear sweep voltammetry (LSV) with bare ITO (black line) and electrodeposition of Ag on ITO in solution (blue line), in agarose hydrogel (red line) from a solution containing 1 mM H_2_O_2_ in phosphate buffer (pH 7.4). Electrodeposition was performed at −0.35 V (vs. Ag/AgCl QRE) for 5 s. The indicated numbers are each onset potential; Figure S4: The size distribution histograms for silver nanoparticles deposited on ITO in solution (a), in agarose hydrogel (b); Figure S5: SEM micrographs of ITO surface upon electrodeposition of silver (a) in solution, (b) in agarose hydrogel at both ×50 k magnification; Figure S6: Anodic stripping voltammetry curve on silver nanoparticles electrodeposited on ITO prepared the same as in Figure 4(b); Figure S7: Calibration plot for the charge values at 5 s in Figure 6(b) against the concentration of H_2_O_2_; Figure S8: Amperometric responses of Ag nanoparticles modified ITO in Ar-saturated phosphate buffer (pH 7.4) with addition of 0.5 mM interference molecules (ascorbic acid, glucose, nitrate) and 0.5 mM H_2_O_2_.

## References

[1] Liu Q., Chen D., Kang Z. (2015). One-step electrodeposition process to fabricate corrosion-resistant superhydrophobic surface on magnesium alloy. ACS Appl. Mater. Interfaces.

[2] Zhao M., Balachandran R., Patterson Z., Gouk R., Verhaverbeke S., Shadman F., Keswani M. (2015). Contactless bottom-up electrodeposition of nickel for 3D integrated circuits. RSC Adv..

[3] Qiu Z., Tang D. (2020). Nanostructure-based photoelectrochemical sensing platforms for biomedical applications. J. Mater. Chem. B.

[4] Ravindran A., Chandran P., Khan S.S. (2013). Biofunctionalized silver nanoparticles: Advances and prospects. Colloids Surf. B Biointerfaces.

[5] Sun X., Zhang X., Ma Q., Guan X., Wang W., Luo J. (2020). Revisiting the Electroplating Process for Lithium-Metal Anodes for Lithium-Metal Batteries. Angew. Chem. Int. Ed. Engl..

[6] Arico A.C., Bruce P., Scrosati B., Tarascon J.-M., Schalkwijk W.V. (2005). Nanostructured materials for advanced energy conversion and storage devices. Nat. Mater..

[7] Pomerantseva E., Bonaccorso F., Feng X., Cui Y., Gogotsi Y. (2019). Energy storage: The future enabled by nanomaterials. Science.

[8] Ren B., Liu G.K., Lian X.B., Yang Z.L., Tian Z.Q. (2007). Raman spectroscopy on transition metals. Anal. Bioanal. Chem..

[9] Ma M., Trzesniewski B.J., Xie J., Smith W.A. (2016). Selective and Efficient Reduction of Carbon Dioxide to Carbon Monoxide on Oxide-Derived Nanostructured Silver Electrocatalysts. Angew. Chem. Int. Ed. Engl..

[10] Liu H., Park J., Chen Y., Qiu Y., Cheng Y., Srivastava K., Gu S., Shanks B.H., Roling L.T., Li W. (2021). Electrocatalytic Nitrate Reduction on Oxide-Derived Silver with Tunable Selectivity to Nitrite and Ammonia. ACS Catal..

[11] Jamkhande P.G., Ghule N.W., Bamer A.H., Kalaskar M.G. (2019). Metal nanoparticles synthesis: An overview on methods of preparation, advantages and disadvantages, and applications. J. Drug Deliv. Sci. Technol..

[12] Cioffi N., Colaianni L., Ieva E., Pilolli R., Ditaranto N., Angione M.D., Cotrone S., Buchholt K., Spetz A.L., Sabbatini L. (2011). Electrosynthesis and characterization of gold nanoparticles for electronic capacitance sensing of pollutants. Electrochim. Acta.

[13] Caschera D., Federici F., Zane D., Focanti F., Curulli A., Padeletti G. (2009). Gold nanoparticles modified GC electrodes: Electrochemical behaviour dependence of different neurotransmitters and molecules of biological interest on the particles size and shape. J. Nanopart. Res..

[14] Baig N., Kammakakam I., Falath W. (2021). Nanomaterials: A review of synthesis methods, properties, recent progress, and challenges. Mater. Adv..

[15] Abbott A.P., Griffith J., Nandhra S., O’Connor C., Postlethwaite S., Ryder K.S., Smith E.L. (2008). Sustained electroless deposition of metallic silver from a choline chloride-based ionic liquid. Surf. Coat. Technol..

[16] Pei A., Zheng G., Shi F., Li Y., Cui Y. (2017). Nanoscale Nucleation and Growth of Electrodeposited Lithium Metal. Nano Lett..

[17] Grujicic D., Pesic B. (2002). Electrodeposition of copper: The nucleation mechanisms. Electrochim. Acta.

[18] Park K., Kim E., Park J.H., Hwang S. (2017). Influence of an active vibration isolator and electrochemical cell design on electrochemical measurements to minimize natural convection. Electrochem. Commun..

[19] Suryanto B.H.R., Gunawan C.A., Lu X., Zhao C. (2012). Tuning the electrodeposition parameters of silver to yield micro/nano structures from room temperature protic ionic liquids. Electrochim. Acta.

[20] Kazeminezhad I., Barnes A.C., Holbrey J.D., Seddon K.R., Schwarzacher W. (2007). Templated electrodeposition of silver nanowires in a nanoporous polycarbonate membrane from a nonaqueous ionic liquid electrolyte. Appl. Phys. A.

[21] Simka W., Puszczyk D., Nawrat G. (2009). Electrodeposition of metals from non-aqueous solutions. Electrochim. Acta.

[22] Abbott A.P., El Ttaib K., Frisch G., Ryder K.S., Weston D. (2012). The electrodeposition of silver composites using deep eutectic solvents. Phys. Chem. Chem. Phys..

[23] Mahinroosta M., Jomeh Farsangi Z., Allahverdi A., Shakoori Z. (2018). Hydrogels as intelligent materials: A brief review of synthesis, properties and applications. Mater. Today Chem..

[24] Herrmann A., Haag R., Schedler U. (2021). Hydrogels and Their Role in Biosensing Applications. Adv. Healthc. Mater..

[25] Tang J.D., Mura C., Lampe K.J. (2019). Stimuli-Responsive, Pentapeptide, Nanofiber Hydrogel for Tissue Engineering. J. Am. Chem. Soc..

[26] Vashist A., Vashist A., Gupta Y.K., Ahmad S. (2014). Recent advances in hydrogel based drug delivery systems for the human body. J. Mater. Chem. B.

[27] Dreiss C.A. (2020). Hydrogel design strategies for drug delivery. Curr. Opin. Colloid Interface Sci..

[28] Khajouei S., Ravan H., Ebrahimi A. (2020). DNA hydrogel-empowered biosensing. Adv. Colloid Interface Sci..

[29] Xu W., Huang L.-B., Wong M.-C., Chen L., Bai G., Hao J. (2017). Environmentally Friendly Hydrogel-Based Triboelectric Nanogenerators for Versatile Energy Harvesting and Self-Powered Sensors. Adv. Energy Mater..

[30] Pernodet N., Maaloum M., Tinland B. (1997). Pore size of agarose gels by atomic force microscopy. Electrophoresis.

[31] Kang H., Hwang S., Kwak J. (2015). A hydrogel pen for electrochemical reaction and its applications for 3D printing. Nanoscale.

[32] Kim B.-K., Park K. (2022). Mass Transport Properties and Influence of Natural Convection for Voltammetry at the Agarose Hydrogel Interface. J. Electrochem. Sci. Technol..

[33] Juven B.J., Pierson M.D. (1996). Antibacterial Effects of Hydrogen Peroxide and Methods for Its Detection and Quantitationt. J. Food Prot..

[34] Wang J., Lin Y., Chen L. (1993). Organic-phase biosensors for monitoring phenol and hydrogen peroxide in pharmaceutical antibacterial products. Analyst.

[35] Lopez-Lazaro M. (2007). Dual role of hydrogen peroxide in cancer: Possible relevance to cancer chemoprevention and therapy. Cancer Lett..

[36] Bautista P., Mohedano A.F., Gilarranz M.A., Casas J.A., Rodriguez J.J. (2007). Application of Fenton oxidation to cosmetic wastewaters treatment. J. Hazard Mater..

[37] Niethammer P., Grabher C., Look A.T., Mitchison T.J. (2009). A tissue-scale gradient of hydrogen peroxide mediates rapid wound detection in zebrafish. Nature.

[38] Ivanova A.S., Merkuleva A.D., Andreev S.V., Sakharov K.A. (2019). Method for determination of hydrogen peroxide in adulterated milk using high performance liquid chromatography. Food Chem..

[39] Abo M., Urano Y., Hanaoka K., Terai T., Komatsu T., Nagano T. (2011). Development of a highly sensitive fluorescence probe for hydrogen peroxide. J. Am. Chem. Soc..

[40] Lee D., Khaja S., Velasquez-Castano J.C., Dasari M., Sun C., Petros J., Taylor W.R., Murthy N. (2007). In vivo imaging of hydrogen peroxide with chemiluminescent nanoparticles. Nat. Mater..

[41] Chen W., Cai S., Ren Q.Q., Wen W., Zhao Y.D. (2012). Recent advances in electrochemical sensing for hydrogen peroxide: A review. Analyst.

[42] Luo Y., Liu H., Rui Q., Tian Y. (2009). Detection of Extracellular H2O2 Released from Human Liver Cancer Cells Based on TiO2 Nanoneedles with Enhanced Electron Transfer of Cytochrome c. Anal. Chem..

[43] Mao L., Osborne P.G., Yamamoto K., Kato T. (2002). Continuous On-Line Measurement of Cerebral Hydrogen Peroxide Using Enzyme-Modified Ring-Disk Plastic Carbon Film Electrode. Anal. Chem..

[44] Yang D., Ni N., Cao L., Song X., Alhamoud Y., Yu G., Zhao J., Zhou H.B. (2019). Silver doped mesoporous silica nanoparticles based electrochemical enzyme-Less sensor for determination of H2O2 released from live cells. Micromachines.

[45] Qin X., Wang H., Wang X., Miao Z., Fang Y., Chen Q., Shao X. (2011). Synthesis of dendritic silver nanostructures and their application in hydrogen peroxide electroreduction. Electrochim. Acta.

[46] Kim B.-K., Seo D., Lee J.Y., Song H., Kwak J. (2010). Electrochemical deposition of Pd nanoparticles on indium-tin oxide electrodes and their catalytic properties for formic acid oxidation. Electrochem. Commun..

[47] Katz E., Willner I., Wang J. (2004). Electroanalytical and Bioelectroanalytical Systems Based on Metal and Semiconductor Nanoparticles. Electroanalysis.

[48] Liu L., Yellinek S., Valdinger I., Donval A., Mandler D. (2015). Important Implications of the Electrochemical Reduction of ITO. Electrochim. Acta.

[49] Kim B.-K., Lee J.Y., Park J.H., Kwak J. (2013). Electrochemical detection of dopamine using a bare indium–tin oxide electrode and scan rate control. J. Electroanal. Chem..

[50] Sandmann G., Dietz H., Plieth W. (2000). Preparation of silver nanoparticles on ITO surfaces by a double-pulse method. J. Electroanal. Chem..

[51] Amatore C., Szunerits S., Thouin L., Warkocz J.-S. (2001). The real meaning of Nernst’s steady diffusion layer concept under non-forced hydrodynamic conditions. A simple model based on Levich’s seminal view of convection. J. Electroanal. Chem..

[52] Bard A.J., Faulkner L.R. (2000). Electrochemical Methods Fundamentals and Applications.

[53] Su C., An M., Yang P., Gu H., Guo X. (2010). Electrochemical behavior of cobalt from 1-butyl-3-methylimidazolium tetrafluoroborate ionic liquid. Appl. Surf. Sci..

[54] Meng F., Yan X., Liu J., Gu J., Zou Z. (2011). Nanoporous gold as non-enzymatic sensor for hydrogen peroxide. Electrochim. Acta.

[55] Anson F.C., Osteryoung R.A. (1983). Chronocoulometry: A Convenient, Rapid and Reliable Technique for Detection and Determination of Adsorbed Reactants. J. Chem. Educ..

[56] Harris D.C., Lucy C.A. (2022). Quantitative Chemical Analysis.

[57] Sophia J., Muralidharan G. (2015). Amperometric sensing of hydrogen peroxide using glassy carbon electrode modified with copper nanoparticles. Mater. Res. Bull..

[58] Kong L., Ren Z., Zheng N., Du S., Wu J., Tang J., Fu H. (2014). Interconnected 1D Co3O4 nanowires on reduced graphene oxide for enzymeless H_2_O_2_ detection. Nano Res..

[59] Brzózka A., Brudzisz A., Jeleń A., Kozak M., Wesół J., Iwaniec M., Sulka G.D. (2021). A comparative study of electrocatalytic reduction of hydrogen peroxide at carbon rod electrodes decorated with silver particles. Mater. Sci. Eng. B.

[60] Dang W., Sun Y., Jiao H., Xu L., Lin M. (2020). AuNPs-NH_2_/Cu-MOF modified glassy carbon electrode as enzyme-free electrochemical sensor detecting H_2_O_2_. J. Electroanal. Chem..

[61] Sheng Q., Shen Y., Zhang J., Zheng J. (2017). Ni doped Ag@C core–shell nanomaterials and their application in electrochemical H_2_O_2_ sensing. Anal. Methods.

[62] Kumar V., Gupta R.K., Gundampati R.K., Singh D.K., Mohan S., Hasan S.H., Malviya M. (2018). Enhanced electron transfer mediated detection of hydrogen peroxide using a silver nanoparticle-reduced graphene oxide-polyanlilne fabricated electrochemical sensor. RCS Adv..

[63] Ngamaroonchote A., Sanguansap Y., Wutikhun T., Karn-Orachai K. (2020). Highly branched gold–copper nanostructures for non-enzymatic specific detection of glucose and hydrogen peroxide. Microchim. Acta.

