# Supplementary material for: Electrodeposition of Silver Nanoparticles on Indium-Doped Tin Oxide Using Hydrogel Electrolyte for Hydrogen Peroxide Sensing
Source: Nanomaterials (Basel). 2022 Dec 22;13(1):48. doi: 10.3390/nano13010048 (PMC9824541; doi:10.3390/nano13010048)
Supplement: Supplementary file 1 [file nanomaterials-13-00048-s001.zip › [SI]_MS.pdf]

Supplementary Material: Electrodeposition of Silver Nanoparticles on Indium-doped Tin Oxide Using Hydrogel Electrolyte for Hydrogen Peroxide Sensing

Jihyeon Kim,[1] Byung-Kwon Kim,[2,*] and Kyungsoon Park,[1,*]

[1] Department of Chemistry and Cosmetics, Jeju National University, Jeju 690-756, Republic of Korea

[2] Department of Chemistry and Nanoscience, Ewha Womans University, Seoul 03760, Republic of Korea

* Corresponding Authors:

kimb@ewha.ac.kr (B.-K.K.); Tel.: +82-2-3277-6578; Fax: +82-2-3277-2385;

kspark895@jejunu.ac.kr (K.P.); Tel.: +82-64-754-3545; Fax: +82-64-756-3561

The effect of pH for silver deposition at agarose hydrogel

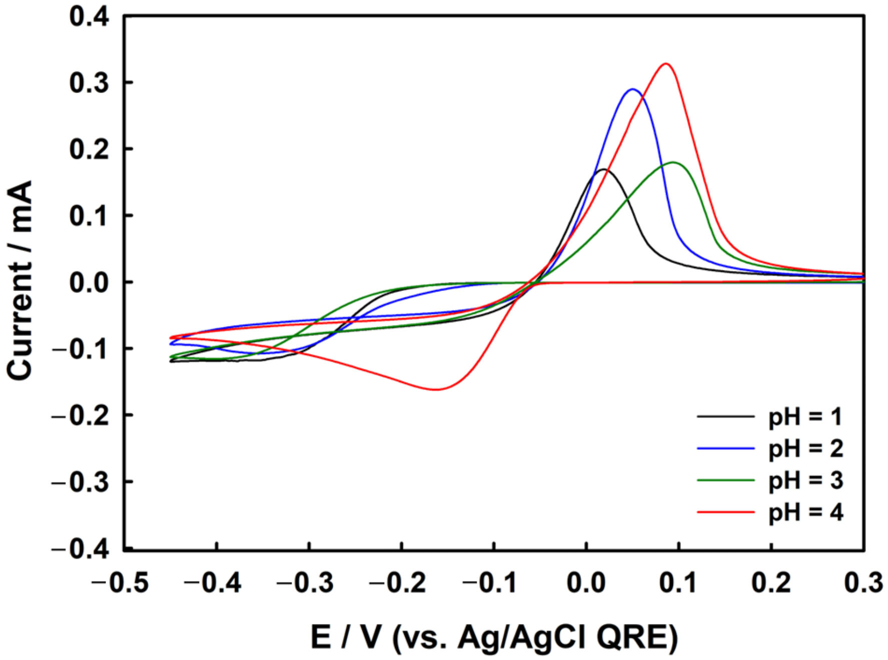

Figure S1. Cyclic voltammetry of silver deposition on ITO using agarose hydrogel (3.2 wt %) as function of pH in 2 mM $AgNO_3$, 1 mM $HClO_4$, and 5 mM $KClO_4$. pH was adjusted by HNO_3. Scan rate: 10 mV/s.

The influnce of the pH condition for silver deposition was obtained by varying the solution pH between pH 1 and pH 4. Increasing the pH from 1 to 4 reveal the change of voltammetric peak current and redox potential at a given scan rate. The maximum peak current and minimum reduction potential of silver deposition found to be at pH 4; pH 4 was selected as the pH of silver electrodeposition for further experiments.

Optimization condition for H_2O_2 sensing : Ag deposition time

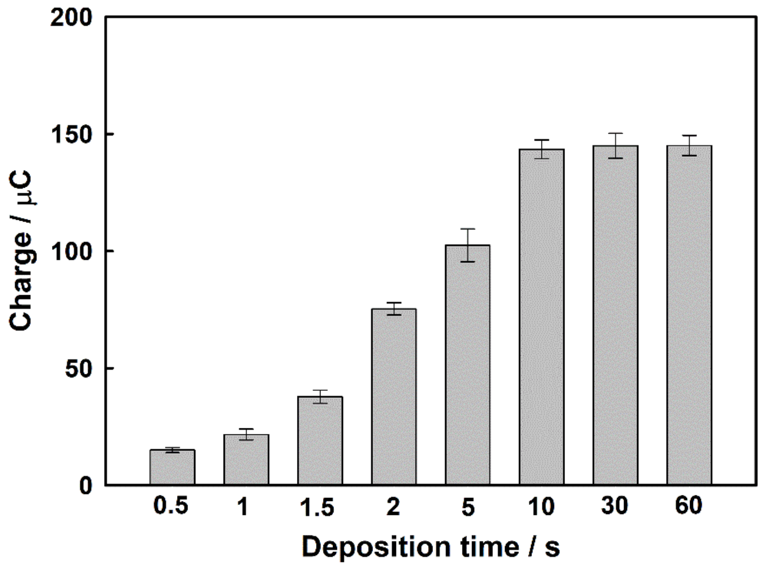

Figure S2. The calibration plot for the H_2O_2 oxidation charge values of chronocoulograms obtained at –0.6 V according to the Ag deposition time. The H_2O_2 sensing experiment conducted in 0.1 M phosphate buffer (pH 7.4) and 700 mM NaCl with 300 μM H_2O_2. The error bars represent the three independent measurements.

Electrochemical analysis of Ag modified ITO

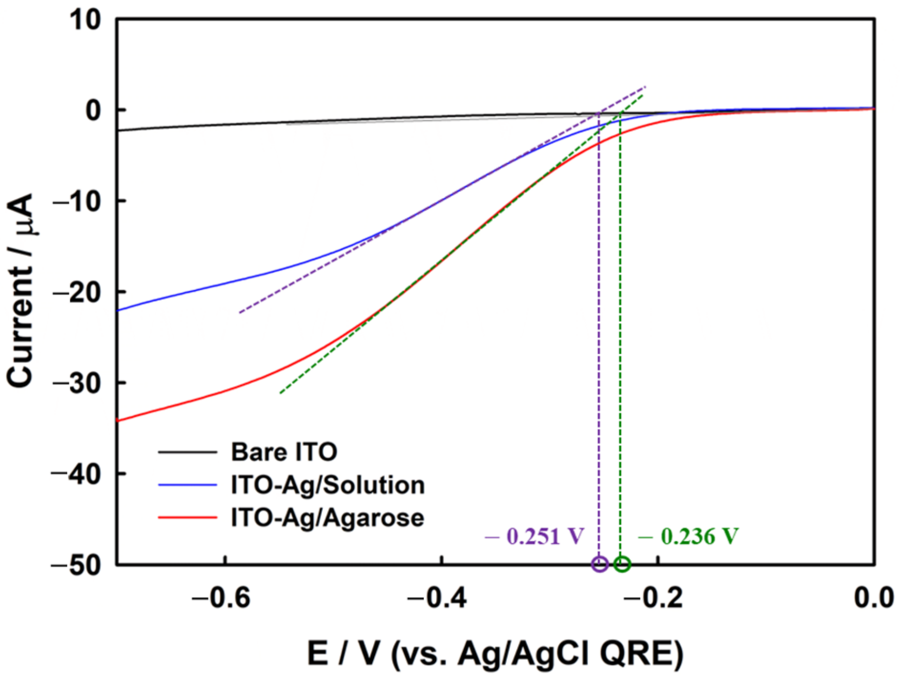

Figure S3. Linear sweep voltammetry (LSV) with bare ITO (black line) and electrodeposition of Ag on ITO in solution (blue line), in agarose hydrogel (red line) from a solution containing 1 mM H_2O_2 in phosphate buffer (pH 7.4). Electrodeposition was performed at –0.35 V (vs. Ag/AgCl QRE) for 5 s. The indicated numbers are each onset potential.

The cathodic onset potential of H_2O_2 in Ag on ITO using agarose (red line, –0.236 V) is higher than that in solution (blue line, –0.251 V). Adsorption of organic compound to the electroactive site (e.g., ankanethiol SAM on electrode) is known to increase the redox potential due to the hindered charge transfer between redox molecules and electrode. From these electrochemical results, we confirmed that the effects of agarose to the H_2O_2 sensing are negligible.

Size distribution for silver nanoparticles on ITO according to the media

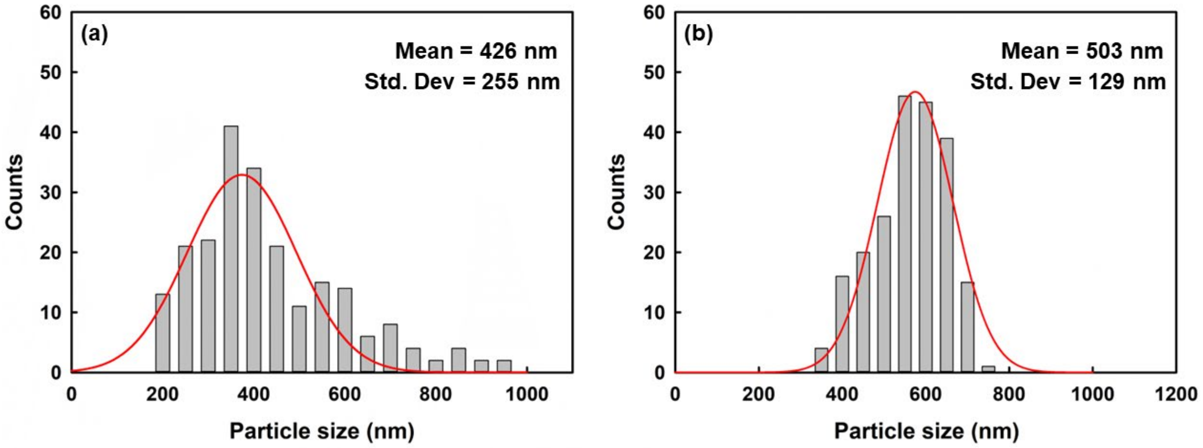

Figure S4. The size distribution histograms for silver nanoparticles deposited on ITO in solution (a), in agarose hydrogel (b).

Figure S4 shows the size distribution histograms of the resulting ITO surface when potential of –0.35 V versus Ag/AgCl QRE are applied for 10 s in the silver-containing solution and agarose hydrogel. Most of silver nanoparticles were size of 200 ~ 800 nm and having a broad size distribution at solution phase deposition in Figure S4(a). However, the majority of the silver nanoparticles produced in agarose hydrogel having a narrow and uniform size distribution ranging from 350 ~ 700 nm compared to the solution phase producing (Figure S4(b)).

Comparison of uniformity of Ag nanocluster on ITO according to the media

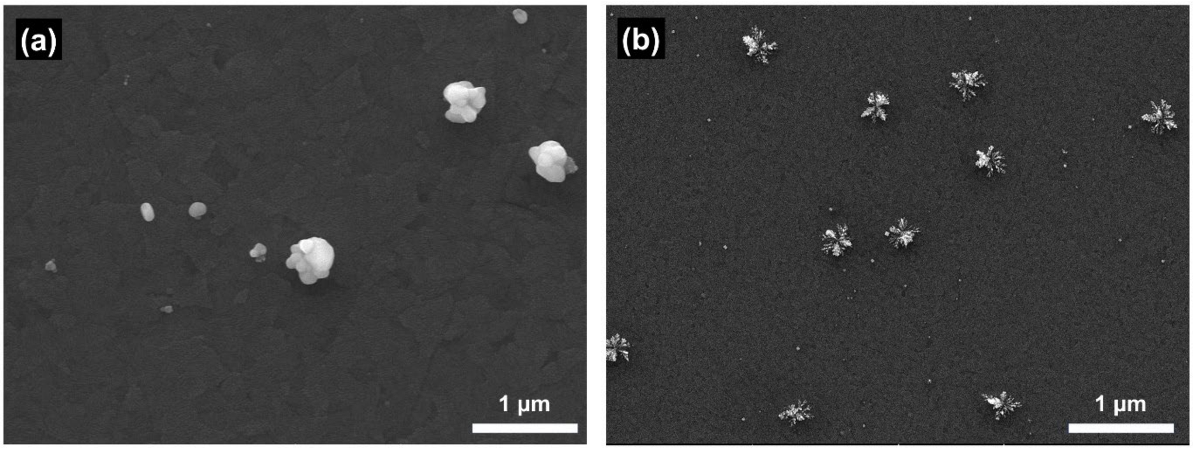

Figure S5. SEM micrographs of ITO surface upon electrodeposition of silver (a) in solution, (b) in agarose hydrogel at both ×50 k magnification.

Electrochemical quantification of silver nanoparticles on ITO

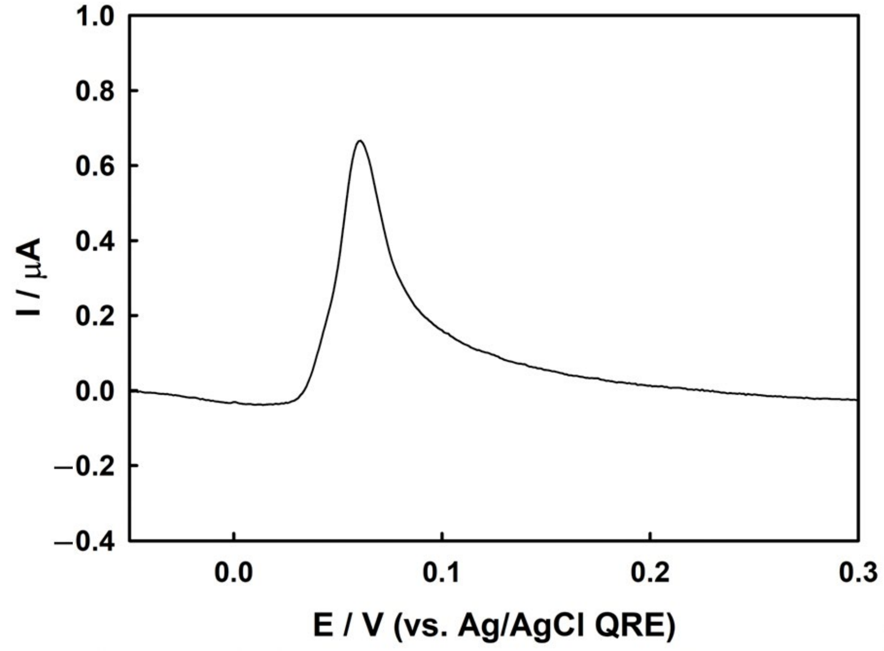

Figure S6. Anodic stripping voltammetry curve on silver nanoparticles electrodeposited on ITO prepared the same as in Figure 4(b).

The baseline corrected total amount of charge during the silver oxidation process is a value of 1.928×10^{-7} C. The quantity of Ag nanoparticles using the charge amount of the anodic current is approximately 7.265×10^{-12} mol/cm^2.

Calibration graph for charge (Q) vs. H_2O_2 concentration

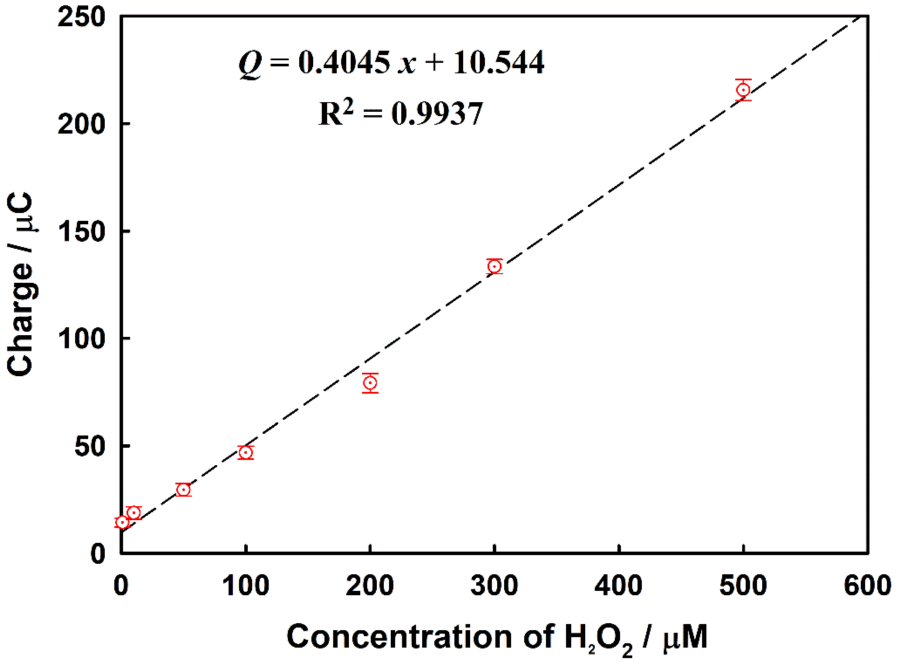

Figure S7. Calibration plot for the charge values at 5 s in Figure 6(b) against the concentration of H_2O_2.

When a calibration curve was drawn using the charge data recorded at 5 s, the slope of the calibration graph is 0.4045 μC/μM and the y-intercept is 10.544 μC. Also, the calibration sensitivity of the sensor is generally determined from the slope of the linear calibration curve.

Selectivity analysis of sensor system using amperometric method

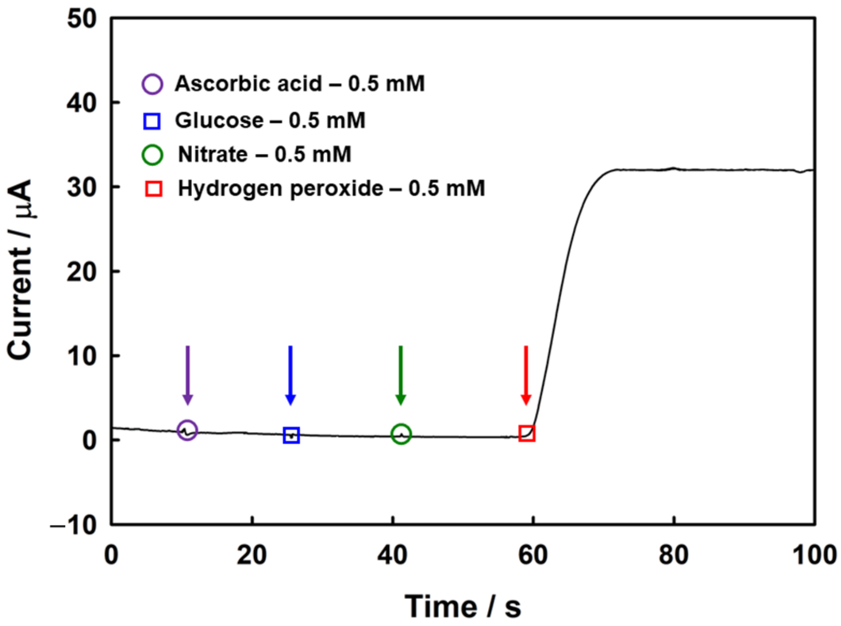

Figure S8. Amperometric responses of Ag nanoparticles modified ITO in Ar-saturated phosphate buffer (pH 7.4) with addition of 0.5 mM interference molecules (ascorbic acid, glucose, nitrate) and 0.5 mM H_2O_2.
