# Supplementary figures and images for: Electrodeposition of Silver Nanoparticles on Indium-Doped Tin Oxide Using Hydrogel Electrolyte for Hydrogen Peroxide Sensing
Source: Nanomaterials (Basel). 2022 Dec 22;13(1):48. doi: 10.3390/nano13010048 (PMC9824541; doi:10.3390/nano13010048)

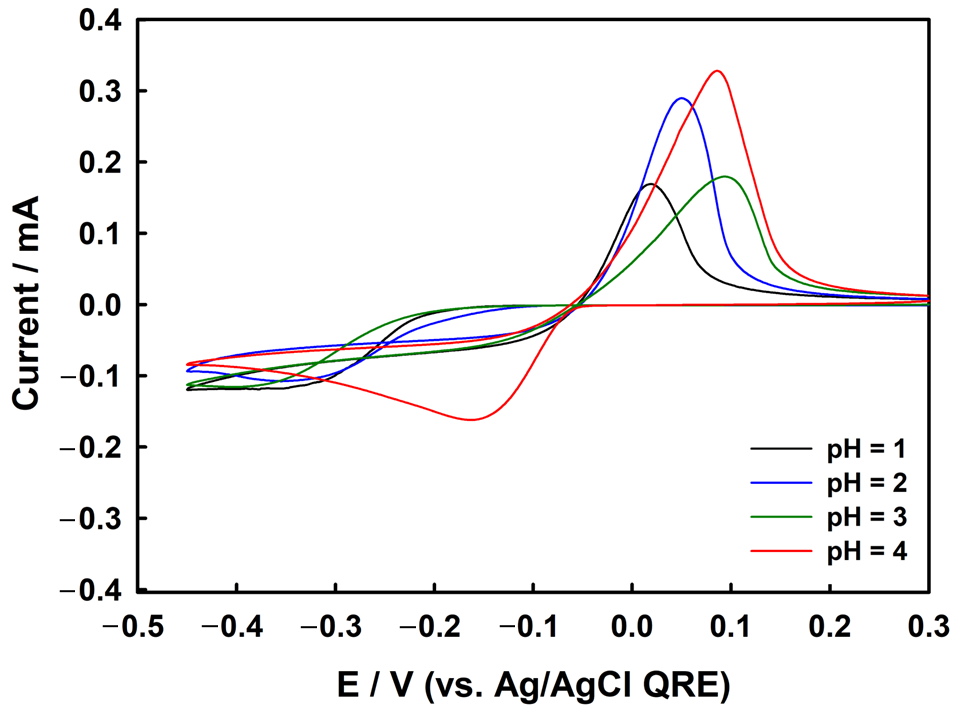

Supplement: Supplementary file 1 [file nanomaterials-13-00048-s001.zip › SI_Figure 1.tif]

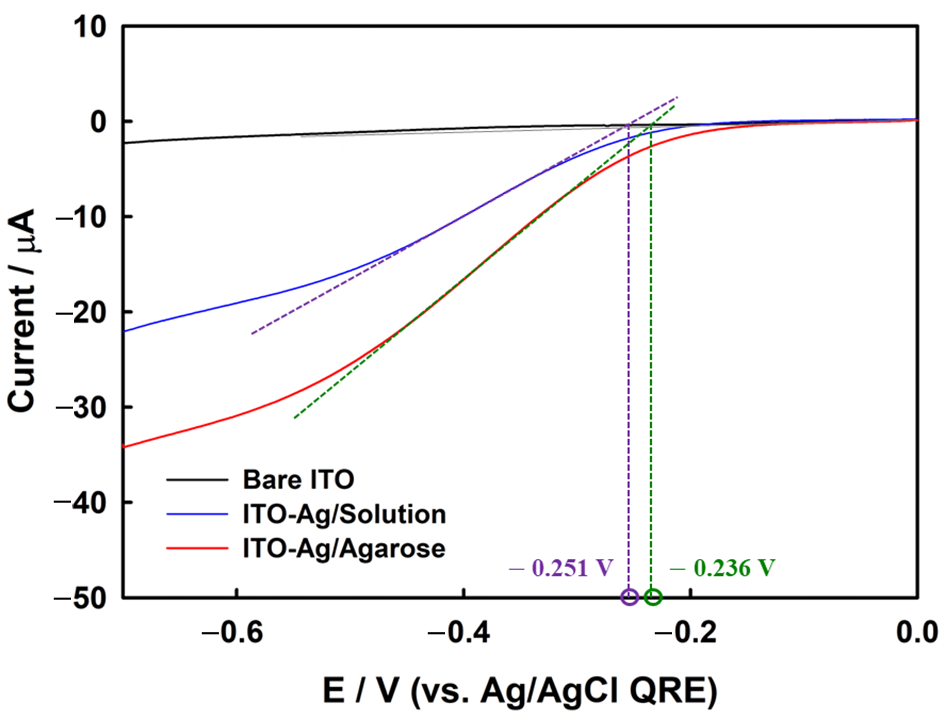

Supplement: Supplementary file 1 [file nanomaterials-13-00048-s001.zip › SI_Figure 3.tif]

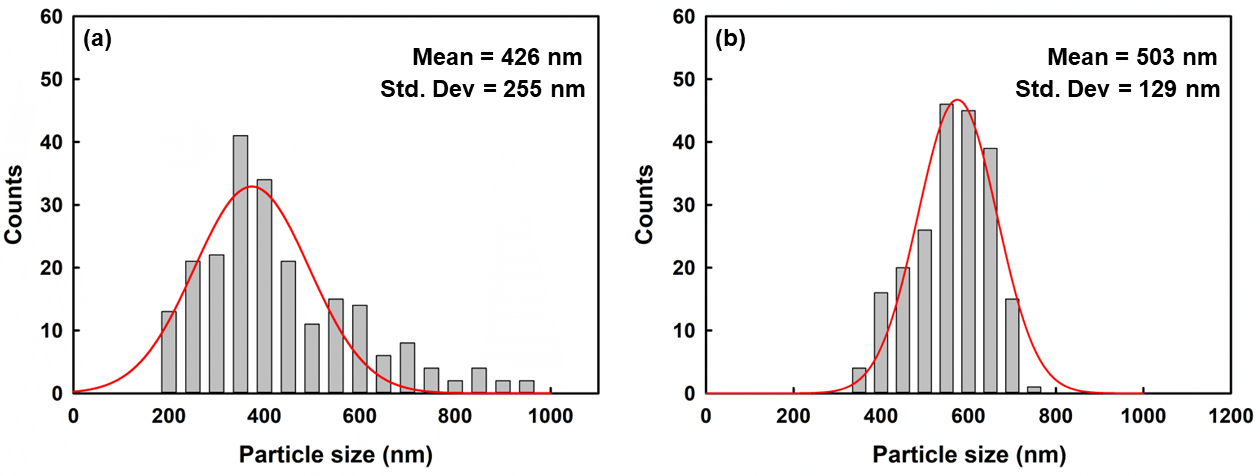

Supplement: Supplementary file 1 [file nanomaterials-13-00048-s001.zip › SI_Figure 4.tif]

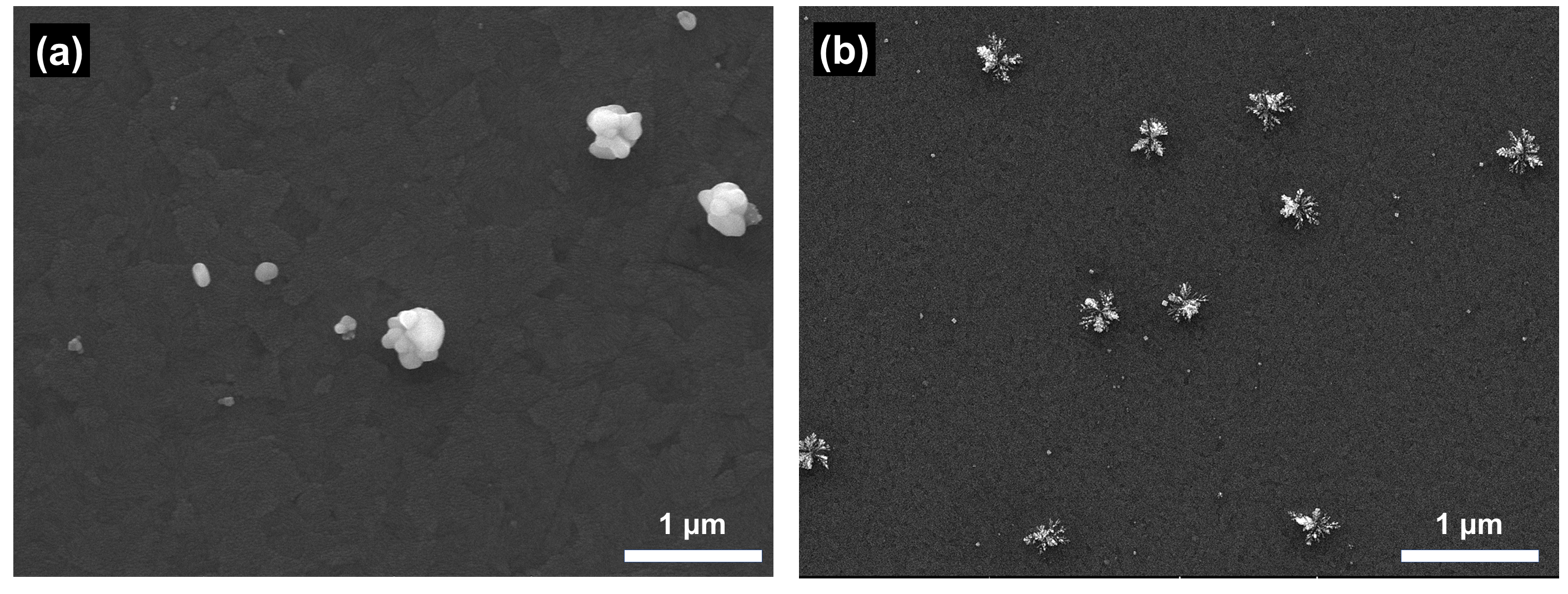

Supplement: Supplementary file 1 [file nanomaterials-13-00048-s001.zip › SI_Figure 5.tif]

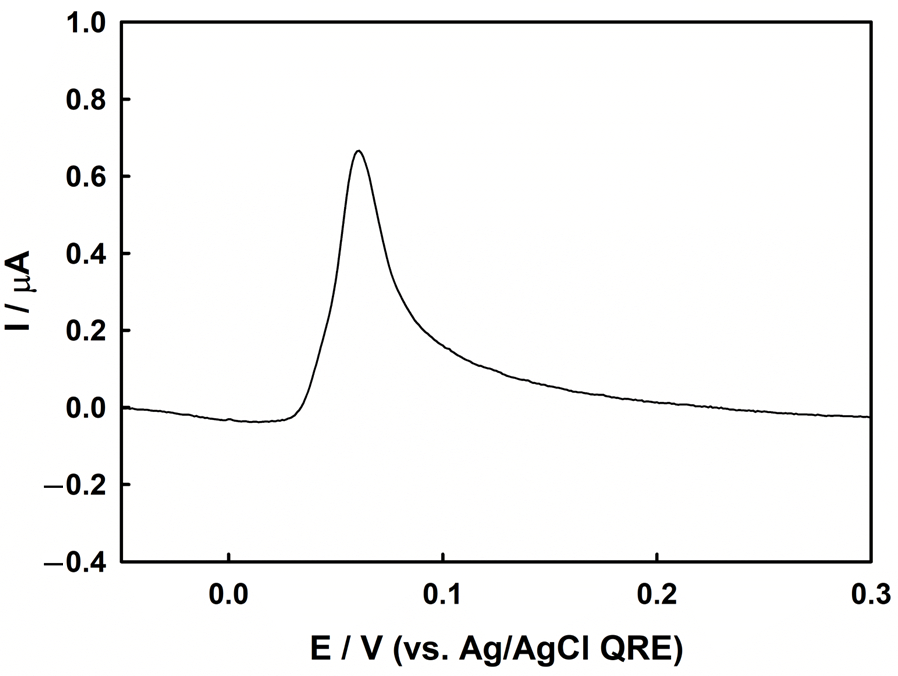

Supplement: Supplementary file 1 [file nanomaterials-13-00048-s001.zip › SI_Figure 6.tif]

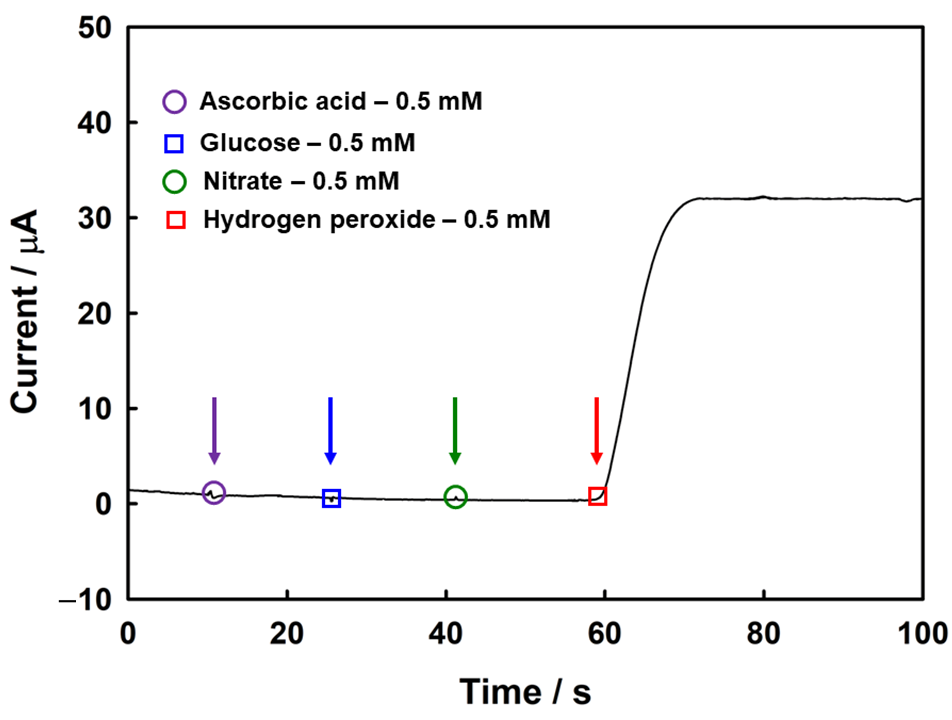

Supplement: Supplementary file 1 [file nanomaterials-13-00048-s001.zip › SI_Figure 8.tif]
